# Profil de la néphropathie diabétique à l’Hôpital Général de Référence Nationale de N'Djamena (Tchad)

**DOI:** 10.11604/pamj.2016.24.193.8415

**Published:** 2016-07-07

**Authors:** Ibrahim Hamat, Guillaume Mahamat Abderraman, Mouhamadou Moustapha Cisse, Mahamat Youssouf, Matar Saboune Djafar, Dionadji Mbainguinam, Tara Fotclossou

**Affiliations:** 1 Unité de Néphrologie Hémodialyse, Hôpital Général de Référence Nationale N’Djamena, Tchad; 2 Service de Néphrologie, Hôpital Aristide le Dantec, Dakar, Sénégal; 3 Service de Médecine Interne, Hôpital de la Renaissance, N’Djamena, Tchad; 4 Unité d’Endocrinologie-Diabétologie, Hôpital Général de Référence Nationale, N’Djamena, Tchad

**Keywords:** Diabète, néphropathie diabétique, N´Djamena, Tchad, Diabetes, diabetic nephropathy, N’Djamena, Chad

## Abstract

**Introduction:**

Le diabète sucré constitue un véritable problème de santé et ses complications touchent plusieurs organes dont les reins. Un diagnostic précoce de la néphropathie diabétique permet de prendre en charge les patients plus efficacement et de façon multidisciplinaire, de retarder sa progression vers l'insuffisance rénale chronique. Peu d'études ont été faites en Afrique dans ce domaine. Au Tchad, nous ne disposons pas de données statistiques sur l'atteinte rénale liée au diabète. C'est pourquoi nous nous proposons d'étudier la néphropathie diabétique avec pour objectifs d'analyser le profil de la néphropathie diabétique des patients de l'hôpital général de référence nationale de N'Djamena.

**Méthodes:**

Nous avions mené une étude transversale descriptive à l'Hôpital Générale de Référence Nationale de N'Djamena d'avril à septembre 2012. Etaient inclus dans l'étude tous les patients diabétiques hospitalisés ou suivis dans les services de néphrologie et d'endocrinologie.

**Résultats:**

Il y avait 54 cas de néphropathie diabétique sur 181 patients soit 29,80%. L'âge de plus de 50 ans représentait 87%, le sexe masculin dominait avec 67%, la durée d'évolution du diabète au moment de la découverte de la néphropathie était de 10,25 ans, la pression artérielle moyenne était de 138 mm Hg, l'HbA1C était supérieur à 6,5% dans 74,5%, l'insuffisance rénale terminale était retrouvée dans 26,90%, une protéinurie à 2,65 g/24 h était détectée dans 60,30%. 57,4% des patients avaient une rétinopathie diabétique au stade III.

**Conclusion:**

Au Tchad, la fréquence de la néphropathie diabétique est de l'ordre de 29,83%. C'est une affection qui touche les hommes avec une moyenne d'âge de 58,7 ans. Le diabète de type 2 est le plus fréquent avec 90,7% des cas. Les facteurs de risque mal contrôlés pouvaient conduire la néphropathie diabétique vers une altération de la fonction rénale notamment l'HTA (70,8%), un déséquilibre glycémique (66,7%) et une protéinurie (62,5%).

## Abstract (en)

**Introduction:**

Diabetes mellitus is a real health problem and its complications affect many organs including the kidneys. Early diagnosis of diabetic nephropathy allows to treat patients more effectively and with a multidisciplinary approach, to slow its progression in chronic renal failure. Few studies have been conducted in Africa in this field of study. In Chad, we have no statistics on renal disease related to diabetes. That is why we propose to study diabetic nephropathy with the aim of analyzing the profile of diabetic nephropathy in patients at the National Reference General Hospital of N’Djamena (CHAD).

**Methods:**

We conducted a descriptive cross-sectional study at the National Reference General Hospital in N’Djamena from April to September 2012. All patients with diabetes hospitalized or treated in the department of nephrology and endocrinology were included in the study.

**Results:**

There were 54 cases of diabetic nephropathy out of a total of 181 patients (29.80%). The patients over the age of 50 years accounted for 87%, masculine gender dominated (67%), diabetes’ progression time at the moment of nephropathy discovery was 10.25 years, mean blood pressure was 138 mm Hg, HbA1c was greater than 6.5% in 74.5%, end-stage renal disease (ESRD) was found in 26.90%, proteinuria 2.65 g / 24 h was found in 60.30%. 57.4% of patients had stage III diabetic retinopathy.

**Conclusion:**

In Chad, the frequency of diabetic nephropathy is approximately 29.83%. This is a condition that affects men with an average age of 58.7 years. Type 2 diabetes is the most common (90.7% of cases). Poorly controlled risk factors might lead to diabetic nephropathy progression in impaired renal function, namely hypertension (70.8%), in glycemic control (66.7%) and in proteinuria (62.5%).

## Introduction

Le diabète sucré constitue de nos jours un vrai problème de santé publique. L'organisation mondiale de la santé (OMS) estime actuellement à 220 millions le nombre de personnes vivant avec cette pathologie dans le monde [1] et ce nombre passerait du simple au double à l'horizon 2030 [2]. Le diabète de type 2 est la forme la plus fréquente et représente environ 90% de toutes les formes de diabète [3]. L'augmentation constante de la fréquence de cette affection pourrait s'expliquer par le changement de mode de vie en particulier la sédentarité, l'alimentation riche en graisse, l'obésité et le vieillissement de la population [4]. La gravité de cette affection est liée à la survenue de complications tant métaboliques, infectieuses que dégénératives parmi lesquelles les manifestations rénales [5]. La néphropathie diabétique (ND) touche 15 à 30% des diabétiques après 10 à 15 ans d'évolution [6]. Elle est l'une des complications les plus fréquentes du diabète et évolue vers l'insuffisance rénale chronique [7]. On la retrouve chez plus de 20% des patients porteurs d'une insuffisance rénale chronique terminale en France [8], 30% au Maroc [9] et 37,8% au Mali [10]. Un diagnostic précoce de cette pathologie permet de prendre en charge les patients plus efficacement et de façon multidisciplinaire, de retarder sa progression vers l'Insuffisance rénale chronique par l'inhibition du système rénine angiotensine (SRA) [11] et par un bon contrôle glycémique, de prévenir ou de limiter l'iatrogénie mais également de traiter les éventuelles complications. Au Tchad, nous ne disposons pas de données statistiques sur l'atteinte rénale liée au diabète. C'est pourquoi nous nous proposons d'étudier la néphropathie diabétique. L'objectif de cette étude est d'analyser le profil de la néphropathie diabétique dans les unités de Néphrologie-Hémodialyse et d'Endocrinologie-Diabétologie de l'hôpital général de référence nationale de N'Djamena.

## Méthodes

C'est une étude transversale descriptive effectuée dans les unités de Néphrologie-Hémodialyse et d'Endocrinologie-Diabétologie de l'hôpital général de référence nationale de N'djamena sur une période de six mois (6) d'avril à septembre 2012. Ont été inclus les patients diabétiques suivis en consultation ou hospitalisés dans les unités sus-citées et répondant à l'un des critères suivants: une hypertension artérielle: elle est définie par une pression artérielle systolique supérieure à 140 mm Hg et/ ou une diastolique supérieure à 90 mm Hg; une rétinopathie diabétique au fond d'œil; une créatininémie élevée: créatininémie supérieure à 14 mg/l (la valeur normale variant entre 7 à 14 mg/l); une protéinurie de 24 heures positive: Si la protéinurie est supérieure à 300 mg/ 24H. Tous les patients présentant les caractéristiques suivantes ont été exclus: hématurie; absence de rétinopathie diabétique au fond d'œil; bilan paraclinique incomplet; absence de consentement. Les données collectées ont été reportées sur une fiche d'enquête individuelle préétablie renfermant l'interrogatoire, l'examen clinique, l'examen biologique et le traitement en cours. Les données recueillies ont été analysées par le logiciel SPSS (Statistics Package Social Sciences version 17). Nous avons eu le consentement éclairé des patients avant leur inclusion dans notre étude. L'identité ainsi que les informations recueillies chez nos patients étaient restées confidentielles.

## Résultats

Etaient inclus dans l´étude tous les patients diabétiques hospitalisés ou suivis dans les services de néphrologie et d´endocrinologie. Il y avait 54 cas de néphropathie diabétique sur 181 patients soit 29,80%. L'âge de plus de 50 ans représentait 87%, le sexe masculin dominait avec 67%, la durée d´évolution du diabète au moment de la découverte de la néphropathie était de 10,25 ans, la pression artérielle moyenne était de 138 mm Hg, l´HbA1C était supérieure à 6,5 % dans 74,5%, l´insuffisance rénale terminale était retrouvait dans 26,90%, une protéinurie à 2,65 g/24 h était détectée dans 60,30%. 57,4% des patients avaient une rétinopathie diabétique au stade III.

**Figure 1:**
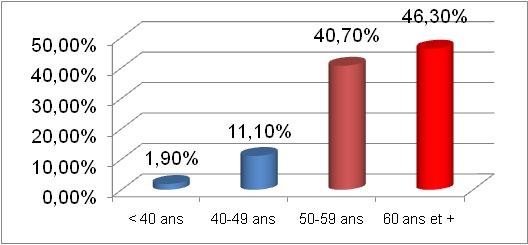
Distribution des patients selon la tranche d’âge

**Age**: La tranche d'âge affectée par la néphropathie diabétique était située entre 50 ans et plus et représentait 87% des cas (Figure 1).

**Sexe**: Le sexe masculin était le plus représenté avec un pourcentage de 63% et le sex ratio H/F était de1, 7.

**Figure 2:**
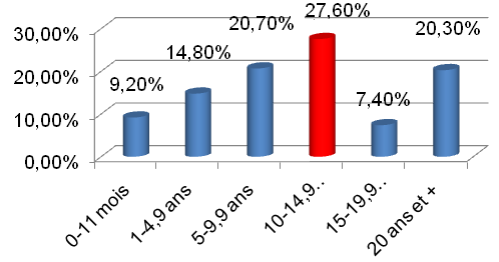
Distribution selon l’ancienneté de diabète

**Ancienneté de diabète**: La durée d'évolution du diabète lors de la découverte de la néphropathie était de 10,25 ans. La tranche la plus représentée était comprise entre 10 et 14,9 ans soit 27,6% (Figure 2).

**Pression artérielle**: Les patients hypertendus représentaient 77,8% des cas, avec une PAM qui se chiffrait à 138 mm Hg.

**Taux d´hémoglobine glyquée**: Un taux d'HbA1c ou hémoglobine glyquée ≥ 6,5% a été noté chez 74,5% de nos patients (Tableau 1).

**Tableau 1:** Répartition des patients selon le croisement entre la TA et l’HbA1c

HbA1c (%)	Tension artérielle (TA)		Total
	TA < 140 mm / 90 mm Hg	TA > 140 / 90 mm Hg	
< 6,5	11(20,3%)	1 (1,9%)	12 (22,2%)
≥ 6,5	1 (1,9%)	41 (75,9%)	42 (77,8%)
**Total**	12 (22,2%)	42 (77,8%)	54(100%)

**Répartition des patients selon la valeur du débit de filtration glomérulaire (DFG)**: Les patients diabétiques avec néphropathie diabétique stade 5 (classification de MORGENSEN) et DFG inférieur 15 ml/mn/1,73 m^²^ SC représentaient 29,6% (Tableau 2).

**Tableau 2:** Répartition des patients selon la valeur du débit de filtration glomérulaire

DFG en ml/mn/1,73m²SC	Nombre	Pourcentage (%)
> 90	**-**	**-**
89 à 60	7	13,0
59 à 30	20	37,0
29 à15	11	20,4
< 15	16	29,6
**Total**	54	100,0

**Valeur de la protéinurie de 24 heures**: Les patients avaient présenté une protéinurie dans 60,3% des cas, avec une protéinurie moyenne qui était de 2,65 g/24 h.

**Résultat de l'examen du Fond d'œil**: Il avait été noté que 57,4% des patients avaient une rétinopathie diabétique au stade III et 42,6% au stade IV (Tableau 3).

**Tableau 3:** Répartition des patients selon les paramètres clinico-biologiques

		Nombre et pourcentage
**Sexe**	Masculin	34 (63%)
	Féminin	20 (37%)
**Tension artérielle**	>140/90 mm Hg	12 (22,2%)
	< 140/90	42 (78,2%
**Hémoglobine glyquée**	≥ 6,5	14 (25,5%)
	< 6,5	40 (74,5%
**Protéinurie des 24H**	>500 mg/24H	21 (39,7%)
	< 500 mg/24H	33 (60,3%)
**Rétinopathie diabétique**	Stade III	23 (42,6%)
	Stade IV	31 (57,4%)

## Discussion

### Age

La tranche d'âge la plus affectée par la néphropathie diabétique dans notre population d'étude était celle à partir de 50 ans et représentait 87% des cas. L'âge moyen de nos patients était de 58,7 ans avec des extrêmes allant de 36 ans à 75 ans. Notre résultat était superposable à une série d'étude réalisée par Taleb et coll. en 2008 au Liban [12] (56,4 ans), Bertal et coll. en 2009 à Marrakech au Maroc (56 ans) [13] et Meisinger et coll. qui trouvent 61,9% en Allemagne en 2008 [14] La prédominance de cette tranche d'âge pourrait s'expliquer par l'importance de la prévalence du diabète de type 2 dont l'âge de survenue est tardif, généralement après 45 ans et une amélioration de la prise en charge des patients.

### Sexe

Notre étude objectivait une nette prédominance masculine avec un pourcentage de 63%. La prédominance masculine a été retrouvée également par Charfi et coll. en 2010 en Tunisie (63,4%) [15] et par Ignatius et coll. au Nigeria en 2009 (52%) [16]. Cette prédominance masculine pourrait s'expliquer par une plus grande accessibilité des hommes dans les structures sanitaires du fait de leur autonomie financière et de leur pouvoir de décision.

### Ancienneté du diabète

La durée d'évolution du diabète lors de la découverte de la néphropathie était de 10,2 ans. Cette durée pourrait s'expliquer par la prédominance du diabète de type 2 (90,7%) qui évolue souvent silencieusement et lentement. Il serait dû également à un accès plus important des patients aux services spécialisés permettant ainsi une meilleure prise en charge, ce qui améliorait leur état de santé et allongerait leur espérance de vie. Selon l'histoire naturelle de la néphropathie diabétique (ND), l'ancienneté du diabète conditionne l'apparition des complications. La protéinurie apparaît après dix ans d'évolution du diabète et 20% des diabétiques type 2 arrivent au stade d'IRCT (insuffisance rénale chronique terminale). Les patients insuffisants rénaux ont généralement une ancienneté du diabète plus longue que chez le groupe sans insuffisance rénale (IR) [17].

### Pression artérielle

L'hypertension artérielle est présente chez 77,8% de nos patients. Bouattar et coll. au Maroc avaient démontré que en 2009, 79,3% des patients de leur étude étaient hypertendus [18]. De nombreuses études montrent que l'hypertension artérielle est l'une des complications du diabète par atteinte vasculaire. L'HTA est connue comme facteur de risque favorisant le développement et l'aggravation de la néphropathie diabétique même à un stade plus précoce [19]. La plupart des études ont confirmé qu'un contrôle optimal de la pression artérielle prévient ou ralentit le développement des lésions rénales au cours du diabète et permet de réduire la morbi-mortalité [20].

### Hémoglobine glyquée

Le résultat de notre étude montrait que 74,5% de nos patients présentent une hémoglobine glyquée dépassant la valeur supposée normale (< 6,5%) traduisant ainsi un déséquilibre glycémique prolongé. Les patients hypertendus avec un déséquilibre glycémique représentait 75,9% des cas. Il existe une relation statistique significative entre le déséquilibre glycémique et l'hypertension tension artérielle (p = 000). Le dosage de l'HbA1c est le principal moyen de surveillance et de jugement de l'équilibre glycémique sur les 3 mois précédents chez les diabétiques. Son dosage trimestriel systématique permet donc un suivi quasi constant de l'équilibre glycémique, de l'efficacité du traitement et éventuellement un ajustement thérapeutique. Le contrôle de l'HbA1c est donc étroitement lié à la prévention des complications micro et macro-vasculaires liées au diabète.

### Clairance de la créatinine

Notre étude montrait que 29,6% de nos patients étaient au stade d'insuffisance rénale chronique terminale. Pouteil-Noble en France en 2001 constatait 20% des cas d'IRCT [8]. Cette différence constatée pourrait s'expliquer chez nos patients par la présence de nombreux facteurs de risque notamment: un mauvais contrôle glycémique (66,7%); une prévalence élevée de l'hypertension artérielle (70,8%) et son mauvais contrôle (41,6% sous traitement antihypertenseur); l'absence systématique de mesure de néphroprotection chez de nombreux patients (37,5% sous IEC ou inhibiteur de l'enzyme de conversion); importance de protéinurie (62,5%). La vitesse de dégradation de la fonction rénale dans la néphropathie diabétique dépend étroitement de la pression artérielle et de la protéinurie. Il existe une corrélation étroite entre le mauvais contrôle tensionnel et la dégradation de la fonction rénale bien documentée dans le diabète. En bloquant le système rénine angiotensine, en contrôlant la tension artérielle et les autres facteurs de risque, on peut ralentir de façon significative la dégradation du DFG à environ 2 à 3ml/mn/an [21].

### Protéinurie de 24 heures

Le résultat de notre étude montrait que 74,5% de nos patients avaient une protéinurie de 24 heures positive. L'incidence de la protéinurie dans notre population d'étude contrastait avec celles d'autres auteurs qui avaient eu des valeurs plus basses. Ainsi, en Iran [14], la protéinurie était rencontrée chez 5,8% des patients dans une étude de Nakhjavani et coll. en 2008. En Allemagne, Meisinger et coll. en 2008 [15] avaient objectivé une protéinurie chez 9,% des patients. En Angleterre, Rury et coll. en 2008 notaient une protéinurie chez 5% des patients [22]. Cette différence constatée pourrait s'expliquer chez nos patients par: l'absence systématique de mesure de néphroprotection (37,5% sont sous IEC ou inhibiteur de l'enzyme de conversion); l'importance du déséquilibre glycémique observée (66,7%); l'importance de l'incidence de l'HTA (70,8%) et son mauvais contrôle (41,6% sous traitement antihypertenseur). Les patients présentant une protéinurie sont exposés à un risque élevé de dégradation rapide du débit de filtration glomérulaire et seule une stratégie de prise en charge intensive peut réduire de moitié les complications micro et macro-vasculaires [22].

## Conclusion

La néphropathie diabétique est une affection dont l'évolution ultime est caractérisée par la survenue d'une insuffisance rénale chronique terminale. Notre travail était une étude transversale descriptive et analytique réalisée dans les unités de Néphrologie-Hémodialyse et d'Endocrinologie-Diabétologie de l'Hôpital Général de référence nationale sur une période de six (6) mois d'avril à septembre 2012 et avait pour objectif de contribuer à analyser l'incidence de la néphropathie diabétique. Notre population d'étude était composée de 181 patients parmi lesquels 54 avaient répondu aux critères d'inclusions. Au Tchad, la fréquence de la néphropathie diabétique est de l'ordre de 29,83%. C'est une affection qui touche les personnes âgées avec une moyenne de 58,7 ans. Elle survient majoritairement chez les sujets de sexe masculin (63%) et est liée essentiellement au diabète de type 2 (90,7%). Les facteurs de risque mal contrôlés pouvaient conduire la néphropathie diabétique vers une altération de la fonction rénale notamment l'HTA (70,8%), un déséquilibre glycémique (66,7%) et une protéinurie (62,5%).

### Etat des connaissances actuelle sur le sujet

- La néphropathie diabétique (ND) touche 15 à 30 % des diabétiques après 10 à 15 ans d'évolution;
- En France [8], il y a 20% d'insuffisance rénale chronique par néphropathie diabétique, 30% au Maroc [9] et 37,8% au Mali [10].

### Contribution de notre étude à la connaissance

- Au Tchad, la prévalence de la néphropathie diabétique est de 29,83%.
